# The Effect of Prebedtime Behaviors on Sleep Duration and Quality in Children: Protocol for a Randomized Crossover Trial

**DOI:** 10.2196/63692

**Published:** 2024-08-20

**Authors:** Rosie Jackson, Chao Gu, Jillian Haszard, Kim Meredith-Jones, Barbara Galland, Justine Camp, Deirdre Brown, Rachael Taylor

**Affiliations:** 1 Department of Medicine University of Otago Dunedin New Zealand; 2 Haszard Biostatistics Dunedin New Zealand; 3 Department of Women's and Children's Health University of Otago Dunedin New Zealand; 4 Department of Psychological Medicine University of Otago Dunedin New Zealand

**Keywords:** screen time, digital device, diet, physical activity, objective measurement, wearable camera, sleep, mobile phone

## Abstract

**Background:**

It is recommended that children should avoid eating dinner, being physically active, or using screens in the hour before bed to ensure good sleep health. However, the evidence base behind these guidelines is weak and limited to cross-sectional studies using questionnaires.

**Objective:**

The aim of this randomized crossover trial was to use objective measures to experimentally determine whether recommendations to improve sleep by banning electronic media, physical activity, or food intake in the hour before bed, impact sleep quantity and quality in the youth.

**Methods:**

After a baseline week to assess usual behavior, 72 children (10-14.9 years old) will be randomized to four conditions, which are (1) avoid all 3 behaviors, (2) use screens for at least 30 minutes, (3) be physically active for at least 30 minutes, and (4) eat a large meal, during the hour before bed on days 5 to 7 of weeks 2 to 5. Families can choose which days of the week they undertake the intervention, but they must be the same days for each intervention week. Guidance on how to undertake each intervention will be provided. Interventions will only be undertaken during the school term to avoid known changes in sleep during school holidays. Intervention adherence and shuteye latency (time from getting into bed until attempting sleep) will be measured by wearable and stationary PatrolEyes video cameras (StuntCams). Sleep (total sleep time, sleep onset, and wake after sleep onset) will be measured using actigraphy (baseline, days 5 to 7 of each intervention week). Mixed effects regression models with a random effect for participants will be used to estimate mean differences (95% CI) for conditions 2 to 4 compared with condition 1.

**Results:**

Recruitment started in March 2024, and is anticipated to finish in April 2025. Following data analysis, we expect that results will be available later in 2026.

**Conclusions:**

Using objective measures, we will be able to establish if causal relationships exist between prebedtime behaviors and sleep in children. Such information is critical to ensure appropriate and achievable sleep guidelines.

**Trial Registration:**

Australian New Zealand Clinical Trials Registry ACTRN12624000206527; https://tinyurl.com/3kcjmfnj

**International Registered Report Identifier (IRRID):**

DERR1-10.2196/63692

## Introduction

Ensuring children and youth receive sufficient good-quality sleep is paramount for optimal health and well-being [1,2]. However, poor sleep health is a major issue worldwide [3], including in New Zealand [4]. Children with poor sleep are at increased risk of excess weight [5], do not do as well at school [6], are less resilient [7], and have poorer mental health [8]. Thus, advisory groups worldwide [9-11] endorse several “sleep hygiene” behaviors that are thought to promote good sleep health, encompassing sleep habits, environments (eg, bedding and temperature), and prebed behaviors (limiting the use of electronic media, vigorous exercise and active play, or the consumption of food in the hour before bed). However, the evidence base illustrating that these strategies actually influence sleep is variable. Strong support exists for some aspects including consistent bed and wake times, age-appropriate sleep duration, bedtime routines, sleep independency, and no bedroom electronics [12,13]. By contrast, evidence supporting any effect of screen time, physical activity, or food intake in the hour before bed is less definitive [12-15].

While a potential relationship between electronic media and subsequent sleep has received the most attention, the evidence on which these guidelines are based is mainly from cross-sectional studies that cannot determine causality [16,17]. The much smaller evidence base of prospective studies reports inconsistent findings, with most research not showing any significant relationship between screen time and sleep [18,19]. Using stronger temporal analyses that examine the effect of screen time on sleep each night have also reported a limited impact of evening screen use [20,21]. Furthermore, most studies to date have used recalls of screen use, which are prone to bias and are likely unable to measure screen time in the modern world [22,23]. While evidence is emerging that has used objective measures of screen time, this has often been restricted to individual devices (predominantly phones) [24,25]. This precludes the measurement of behaviors including multitasking where more than 1 device is used simultaneously [26,27]. A further limiting aspect is that most studies have examined the impact of whole-day screen time, rather than that more immediate to bedtime, thought to be more detrimental to sleep [16,28]. Although some, but not all, questionnaire-based analyses by Hartley et al [29] and Belmon et al [30] have reported that the use of evening electronic media was associated with poorer sleep, objective measurement of bedtime smartphone usage (tracking app) did not support these findings [24].

A recent review suggested that interventions to reduce screen time can improve sleep. However, there are many issues with this literature. Interventions have often included day screen use [31], or not specified evening screen use only [32], or included changes to other behaviors that might also influence sleep such as diet and exercise [31]. Much of the assessment has also been through questionnaires, which have produced conflicting findings [33,34].

Although daytime physical activity is beneficial for evening sleep in some studies [35-38], how physical activity before bedtime impacts sleep (the actual recommendation) in youth is unclear, with very limited data. Matsuyama et al [39] showed no difference in accelerometer-measured physical activity of any intensity between those who went to bed earlier versus later. However, the period examined (after school until bedtime) was considerably longer than the guidelines (1-2 hours before bed), and sleep duration was not examined. While others have reported that youth with morning chronotypes tend to have higher levels of physical activity and lower levels of sedentary behavior [40], such data do not analyze a direct relationship between evening physical activity and subsequent sleep. Presumably, the recommendation about evening activity was developed based on experimental work in adults. However, examination of that research also suggests that evening exercise of moderate to vigorous intensity either has no detrimental effect, or is even protective, improving a range of sleep health outcomes [41-44]. Comparable findings have also been reported in large observational studies using objective measures of both behaviors [45]. Given the dearth of research on youth, who may respond differently to adults, experimental evidence directly designed to answer whether evening physical activity impedes sleep is required.

The final recommendation refers to avoidance of large amounts of food close to bedtime. However, as with screens and activity, relatively few studies appear to have investigated associations between the timing of the evening meal (hereafter referred to as dinner) and sleep outcomes in youth. Cross-sectional data in children show that later dinner meals are associated with parental-reported later bedtimes [46] and shorter sleep duration [47]. However, objective measures of sleep have produced mixed results, with studies showing associations between later evening mealtimes and shorter total sleep time (TST) [48] or later sleep midpoint [49], or no difference in sleep duration between early and late dinner eaters [50]. To date, no experimental evidence analyzing meal timing and sleep appears to have been conducted in youth, although comparable data show no effects in adults [51].

Thus, it appears that very little experimental evidence has directly examined whether any of these prebedtime behaviors adversely affect sleep; critical data for producing evidence-based sleep hygiene guidelines. This is particularly relevant considering that implementation of such guidelines might be challenging for families. For example, both parents and children may find the current blanket ban on screen time before bed difficult, if not impossible, to implement regularly, despite more than half of all parents viewing excessive screen time as their top health concern [52]. Given almost ubiquitous engagement in evening screen use by adolescents [53] and the potential for further stress and conflict in families from screen time “bans” [54,55], an examination of the practicality of such guidelines seems warranted. Encouraging children not to eat late or be physically active in the evenings might also be challenging, given they do not see these behaviors as important for sleep [56]. Whether parents also find the recommendations to restrict food and vigorous activity before bedtime difficult, appears not to have been examined. It is also important that sleep hygiene recommendations do not harm children. For example, given that inadequate physical activity is a major issue for children worldwide [57], restricting children from being active close to bedtime is a misguided effort to improve sleep, and may have unintended consequences on overall activity. Such analyses do not appear to have been examined.

Therefore, the primary aim is to use objective measures to experimentally determine whether screen time, physical activity, or a meal in the hour before bed affects TST.

The secondary aims are to determine (1) whether screen time, physical activity, or a meal in the hour before bed affects sleep onset, wake after sleep onset (WASO), shuteye latency, sleep onset latency, sleep disturbances, and sleep impairment; (2) whether screen time, physical activity, or a meal in the hour before bed affects health-related quality of life; (3) whether restricting physical activity before bed influences physical activity during the day; and (4) how participants and families found the interventions and how realistic they considered them to be.

## Methods

### Study Design

The helpful activities before it is time to sleep (HABITS) study is a randomized crossover trial to experimentally determine whether banning electronic media, physical activity, or food intake in the hour before bed actually impact children’s sleep quantity and quality. Wearable cameras will be used to obtain objective measures of prebed behaviors and accelerometers for objective measures of sleep. The study will be conducted in participants’ homes and at the University of Otago in Dunedin, New Zealand.

**Figure 1:**
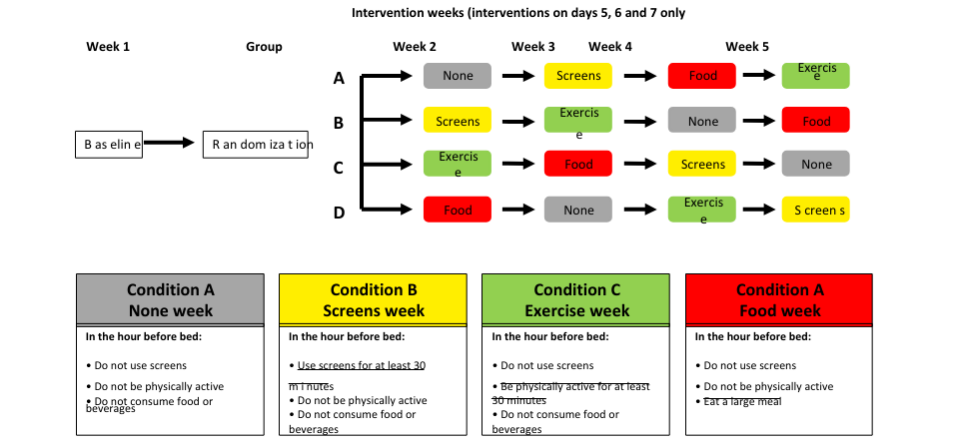
The helpful activities before it is time to sleep study design using Williams Latin square randomization design.

All children will undergo a baseline week to establish usual prebedtime behavior and set bedtimes for the intervention weeks. Each participant will follow 4 experimental prebedtime conditions in a randomized order (Figure 1). The comparison or ideal week will be condition A. In condition B (screens week), participants can use any device and undertake any screen behavior. In condition C (exercise week), suitable options will be suggested for both indoor (eg, wrestling and dancing) and outdoor (tag, sports, and walking) activities. In condition D (food week), participants will be asked to eat a large meal in the hour before bed.

Families will follow each intervention condition on the same consecutive 3 days of each intervention week (days 5-7). In total, 3 days will provide sufficient data to determine how each behavior influences sleep, allows a washout between each condition, and is a pragmatic choice to limit participant or researcher burden. As we are examining the effect of the condition on sleep the same night, this amounts to 3 observations/participant/condition, which should give more reliable estimates than 1 observation or condition. Each family can choose what days of the week are days 5-7 for them, as long as the same days apply across all 5 weeks. All interventions will occur during the school term to limit known changes in sleep caused by holidays [58]. Daylight savings will be avoided and lunar patterns will be recorded to examine the potential for confounding across the different phases.

### Ethical Considerations

The HABITS study was approved by the University of Otago Health Ethics Committee (H23/039) and is registered with the Australian New Zealand Clinical Trials Registry (ACTRN12624000206527; www.anzctr.org.au). Written informed consent will be obtained from adult and child participants before the first appointment. We also follow ethical frameworks for the use of wearable cameras in research [59]. In brief, this includes discussing with participants and families about privacy considerations (eg, everyone in the household needs to be happy to be recorded, remove the camera when toileting or someone asks), families get to view the footage before the research team does and can delete any footage without explanation, and that footage is deleted from cameras as soon as it is uploaded to our high-capacity secure storage system.

### Sample Size

To detect a difference of 15 minutes (assuming within-person SD of 35 minutes) in TST [60], with 90% power at a .05 α level, a sample size of 60 participants would be required. We will recruit 72 children, allowing for 20% nonadherence, dropout, or incomplete data.

### Recruitment and Eligibility

#### Overview

Recruitment will take place through social media and community networks. Interested families will complete an eligibility screening questionnaire online (assessing whether their child has, or previously had, a sleep disorder, any medications that may impact sleep, and ability to undertake physical activity), which they will access through local Facebook (Meta) pages or email from the researchers. Children will be eligible to participate if they are 10-15 years old and live in the Dunedin area. Children will be excluded if they have been diagnosed with a sleep disorder or issue or are on medication that may affect sleep. Children who are not eligible will be sent a standardized text or email by their caregiver. We aim to recruit a sample that is broadly representative of the New Zealand population [61].

Before attending the baseline appointment, potentially eligible participants will be asked to select 3 consecutive nights in the week that would work best for them to complete the intervention (eg, Mondays, Tuesdays, and Wednesdays). These days will be classified as days 5-7 for participant baseline (week 1) and intervention (weeks 2-5) weeks. The researchers will then book their baseline appointment on day 1 of the baseline week.

#### Baseline (Week 1)

At the baseline appointment, researchers will explain the study protocol to potential participants and their caregivers (mainly parents), with opportunities for questions throughout. If the child and their caregiver indicate that they would like to be involved, they will be given a paper version of the assent (child) and consent (caregiver) form to sign. Once consent and assent are signed, caregivers will complete an online demographics questionnaire assessing the child’s date of birth, household address, and ethnicity. Weight (using Tanita electronic scales HD351) and height (using Wedderburn Portable Height Rod, WS-HRP) will be measured by trained researchers using standard procedures [62].

Participants will be given an accelerometer (AX3 [Axivity]) to wear on their nondominant wrist for 7 nights, 24 hours per day, to measure usual sleep (more details are discussed in the *Intervention Adherence* section). To determine usual bedtime practices in terms of screen time, physical activity, and food consumption, participants will be asked to wear a wearable video camera from 90 minutes before bedtime until bedtime on their 3 “intervention” nights (more details are discussed in the *Intervention Adherence* section). A second stationary camera in the child’s bedroom will face the bed and allow researchers to measure in-bed electronic use or eating, and to measure when the participants attempt sleep (shut-eye time; more details are discussed in the *Sleep* section). Participants will be asked not to change their behavior during the baseline week. Information from the cameras will also be used to give researchers insight into how the participants might be able to adapt their nighttime behavior to complete the intervention weeks. Researchers will talk to participants (and families) at the baseline visit about their usual eating, physical activity, and screen time habits to help develop personalized suggestions for completing the intervention weeks.

### Randomization

#### Overview

Once the baseline appointment is completed, participants will be randomized using the REDCap (Research Electronic Data Capture; Vanderbilt University) randomization module [63] to one of 4 intervention group orders using a computer-generated sequence, stratified by age (from 10 to less than 13 years or from 13 to 15 years), with random block sizes of 4 or 8. The 4 group orders are from a Williams Latin square balanced for first-order carryover effects. Usual bedtimes will be discussed with families, based on camera footage from baseline. While bedtime for each night of intervention (ie, nights 5-7 of each intervention week) can be different, each day of the week must be consistent between all 4 intervention weeks.

#### Intervention (Weeks 2-5)

At the start of each intervention week, a trained researcher will visit the participants at home to inform them which intervention (screens, physical activity, food, or none week) they are completing that week. The researchers will discuss potential ideas with the participants based on their baseline week information on how to adhere to that week’s protocol.

As the participants will potentially be asked to do less (ie, not use screens) or more (ie, eat dinner close to bedtime) than they usually would do before bed, they will be given ideas for filling in the time while avoiding their usual prebedtime activities. For example, researchers will suggest games or reading for the nonscreen weeks and discuss rescheduling of the family dinner during the food week. These strategies will be put in place specifically to encourage adherence, so that we can determine the true effect of each prebed behavior on sleep. During the physical activity week, we will give each participant a Fitbit Inspire 3 (Google) to measure their heart rate while exercising. Participants will be able to see their heart rate and asked to aim for a target heart rate of 65% of their maximum ([208-0.7*age]*0.65) [64] during the 30 minutes of activity. Participants and their caregivers will receive regular text reminders, letting them know what intervention to complete each night, and when to turn the cameras on.

### Intervention Adherence

Objective and subjective measures will be used to assess adherence. On days 5-7 of each intervention week, participants will wear a PatrolEyes Max (StuntCams) camera on a chest harness from 90 minutes before bedtime until bedtime. These cameras (weighing 160 g and having dimensions 79×57×27 mm) provide time-stamped footage, face outwards, record audio and video (including overnight use through infrared), and quantify when and how much screen use has occurred [65]. We will also be able to determine when and what food or beverages were consumed, supplemented with a brief questionnaire (text message) on mornings 6, 7, and 8, asking them whether they consumed any food or drinks in the hour before bed last night, as well as any caffeine intake. We will use the wearable cameras to code for moderate-to-vigorous physical activity (Compendium of Physical Activity metabolic equivalent values >4 [66]) using a reliable video coding protocol that codes free-living physical activity behavior, supplemented with information from heart rate monitors and accelerometers. Since the wearable cameras can be removed before bedtime (eg, bathing), a second stationary camera will be set up in the participant’s bedroom 10 minutes before bedtime. This “back-up” camera would detect any time-stamp missing from the wearable camera (as long as the participant is in their bedroom) and is also used to assess some aspects of sleep (more details are discussed in the *Sleep* section). It will not be possible to blind coders (as they are looking for specific behaviors) or participants (as they know what behaviors they need to do), but statistical analysis will be undertaken blinded to the intervention order.

The following predefined cut-offs will be used to classify participants as “adherent” for the per-protocol analysis (ie, those who followed the HABITS protocol):

1. Condition A: children use screens and engage in physical activity for less than 5 minutes and do not consume any food or beverages other than water in the hour before bed.
2. Condition B: children spend at least 30 minutes on screens in the hour before bed (and engage in physical activity for less than 5 minutes and do not consume any food or beverages other than water).
3. Condition C: children spend at least 30 minutes being physically active (light, moderate, or vigorous activity) in the hour before bed (and use screens for less than 5 minutes and do not consume any food or beverages other than water).
4. Condition D: children consume their dinner meal in the hour before bed (and use screens and engage in physical activity for less than 5 minutes).

Video data will be examined to describe how children are spending this time, based on a reliable (κ≥0.8) coding schedule previously developed by our team [65].

### Outcome Measures

#### Sleep

**Figure 2:**
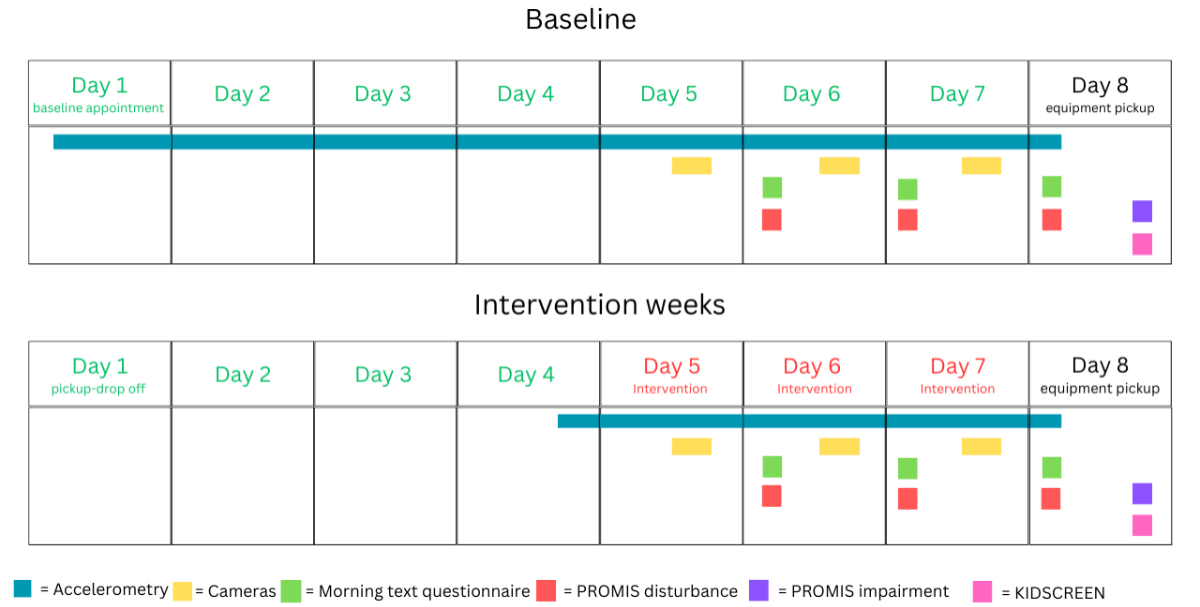
Outline of the measurement schedule. PROMIS: Patient-Reported Outcomes Measurement Information System.

The primary outcome is TST, with secondary outcomes of sleep onset, WASO, shuteye latency, sleep onset latency, sleep disturbances, and sleep impairment assessed using objective and subjective measures (Figure 2). AX3 accelerometers will be worn on the nondominant wrist, 24 hours a day, for 7 days during the baseline week and 3 days during intervention weeks 2-5. These accelerometers provide accurate measures of our sleep variables in this age group, as determined against the gold standard polysomnography [67]. These devices are smaller than a watch, waterproof, unobtrusive, and do not need to be removed for sport or bathing, ensuring high wear time compliance rates [68]. After initialization of the accelerometer, the data will be downloaded with OmGui software (version 1.0.0.30; Open Movement), saved in raw format as .cwa files, then converted into ActiGraph counts for data processing [69]. Sleep variables are obtained using an automated script developed in MATLAB (MathWorks) that uses a count-scaled algorithm to estimate sleep and wake epochs for each individual for each day [70]. Sleep onset is then determined as the start of the first 15 continuous minutes of sleep preceded by 5 minutes of awake. Sleep offset is determined as the last of 15 continuous minutes of sleep followed by 5 minutes of awake. WASO is determined as the number of minutes from the sum of all movement epochs that occur over 5 continuous minutes of awake between sleep onset to offset. TST is determined as the number of minutes from sleep onset to sleep offset minus WASO. The remaining sleep variables of interest will be measured from the stationary video data. Bedtime is determined as the first time the participant gets into bed and under the covers. Shuteye time is determined as the first time the participant stops interacting with any device or person and appears to close their eyes to try and go to sleep. Shuteye latency is calculated as the difference between bedtime and the first shuteye time. Sleep latency is calculated as the difference between shuteye time from the video camera data until sleep onset time from the accelerometer. Sleep disturbances (difficulties falling and staying asleep) and sleep impairment (daytime alertness and sleepiness) will be measured using 2, 8-item PROMIS (Patient-Reported Outcomes Measurement Information System) questionnaires [71]. The questionnaires were originally designed with a 7-day recall and will be used in this context to assess usual sleep disturbances and impairment during the baseline week on day 8 (therefore covering the previous week). An adapted version assessing the previous night only, will be used to measure sleep disturbances on the previous night and will be administered on the morning of days 6, 7, and 8 of each intervention week, asking about “Last night…” rather than “Over the past 7 days.” An adapted version assessing sleep impairment over the previous 3 days will be administered on the evening of day 8 of each intervention week, with the anchors for these questions changing from “Over the past 7 days” to “Over the past 3 days.”

#### Health-Related Quality of Life

Participants will complete the 10-item KIDSCREEN questionnaire that assesses health-related quality of life over the past week [72] at the end of the baseline week. As with the PROMIS impairment questionnaire, an adapted version of the KIDSCREEN questionnaire will be completed in the afternoon of day 8 of each intervention week. The adapted version changes the answer anchors from “Thinking about the last week...” to “Thinking about the last 3 days...”

#### Participant and Family Experiences

At the end of each intervention week, participants and families will provide brief feedback on their experiences, rating the ease of activity on a scale of 1 to 5, and providing reasons for their rating. In addition, 16 participants and families will take part in semistructured interviews upon study completion, offering detailed qualitative feedback on each intervention activity, its feasibility for integration into daily routines, and any changes in sleep resulting from the activities.

### Statistical Analyses

Siblings in the same family may wish to participate, and they would be convenient participants, given their familiarity with the intensive intervention protocols. However, siblings represent family clustering that must be statistically accounted for. We examined the intraclass correlation coefficient (ICC) for families in our recent observational repeated-measures study of screen use and sleep in teens [73], which was low (ICC=0.005). Using this ICC and an average family cluster size of 2, the design effect is 1.005 [74], which has a negligible effect on the required sample size (60*1.005=60.3). Therefore, siblings may be recruited but will be assessed at different times throughout the year, will be individually randomized, and will contribute to no more than half the sample (ie, n=36 participants must not have siblings in the study).

Primary statistical analyses will be undertaken using data from adherent days; secondary analyses will use all data. Mixed effects regression models with a random effect for participants nested within a family cluster will be used to estimate mean differences, 95% CI, and *P* values for conditions 2, 3, and 4 compared with condition 1. Residuals will be plotted and visually assessed for homoskedasticity and normality. Adjustments for within-person confounding (eg, lunar phases) will be undertaken as sensitivity analyses. Results will be reported in line with the CONSORT (Consolidated Standards of Reporting Trials) statement extension to crossover trials. Descriptive analyses will also be undertaken to explore participants’ experiences with the interventions as well as their adherence. Relationships between sleep and quality of life measures will be assessed using repeated measures mixed effects analyses.

## Results

Data collection commenced in February 2022, and the first results are expected to be submitted for publication in 2026.

## Discussion

Ensuring children receive enough good-quality sleep is paramount, given the number worldwide who do not get sufficient sleep for their needs [3]. In fact, it has been argued that addressing sleep health may be a viable way of reducing many health inequities [3]. Therefore, ensuring suitable guidelines exist that are both evidence-based and achievable for families is critical to ensure health impact. At present, we do not have strong evidence that using screens, being physically active, or eating the dinner meal in the hour before bed even impact sleep. In addition, we know that there are many barriers to implementing these behaviors in households. Based on the limited experimental evidence available, we believe that these 3 behaviors might have a limited effect on sleep outcomes in children. It is possible that some, particularly physical activity before bed, might even be beneficial for sleep.

Our proposed trial has several strengths. We have chosen a strong experimental design, are using objective measures of key behaviors to overcome many of the limitations of the current evidence base, and have adequately powered our study to answer our research questions of interest. The HABITS study has been specifically designed to show if these prebedtime behaviors truly influence sleep so that the relevant sleep guidelines can be evidence-based. However, it also has some potential limitations. We will need to ensure that we can recruit a diverse sample, to enable generalizability of findings to a broad demographic. Ideally, we would assess each behavior over the course of a full week, but this was considered inappropriate because of respondent burden. There will be some variation in how families interpret the evening meal and exercise routines suggested, but this should reflect the real world and is appropriate for our pragmatic trial.

## Appendix

Multimedia Appendix 1 Peer-review reports from the Te Kaunihera Rangahau Hauora o Aotearoa / Health Research Council of New Zealand.

## Abbreviations

CONSORT: Consolidated Standards of Reporting Trials
HABITS: helpful activities before it is time for sleep
ICC: intraclass correlation coefficient
PROMIS: Patient-Reported Outcomes Measurement Information System
REDCap: Research Electronic Data Capture
TST: total sleep time
WASO: wake after sleep onset

## Data Availability

The deidentified data sets used or analyzed during this study will be available from the corresponding author on reasonable request.
