# Supplementary material for: The Effect of Prebedtime Behaviors on Sleep Duration and Quality in Children: Protocol for a Randomized Crossover Trial
Source: JMIR Res Protoc. 2024 Aug 20;13:e63692. doi: 10.2196/63692 (PMC11372319; doi:10.2196/63692)
Supplement: Multimedia Appendix 1 [file resprot_v13i1e63692_app1.pdf]

Applicant peer review report

Reviewer # 37

Proposal details

Title	Whakatipu Rakatira: improving sleep as a vehicle to grow healthy future leaders
First named investigator	Professor Rachael Taylor (University of Otago)

Rationale for research Score: 5

This proposal aims to intervene with regards to sleep health in Māori youth, with four projects designed to support this aim.The investigators propose interventions to improve sleep in Māori youth with the hope that the improvements in sleep will cascade into physical and mental health outcomes, addressing the gap in health and achievement outcomes in the Māori. This is a worthwhile endeavour and sleep represents an important intervention target.Therefore, the grant addresses a significant health issue.

Despite these strengths, some issues exist with regards to the proposal. For one, the grant does not effectively incorporate current knowledge into the design of their interventions and at times the proposal is vague. It is also unclear whether there will be new knowledge gained from this proposal.

Design and methods Score: 5

The study design is feasible. The team have extensive experience working with the Māori community and are well positioned to leverage their findings into policy change.

However, I have significant reservations about the study design. Specific comments for each project below:

Project 1: The authors recruit pregnant women and follow-up infants and families for 6 months. Sleep based Interventions in infants are not recommended prior to the 4th month due to the developmental needs of the infant to feed regularly etc .. Therefore, I am unsure whether the timing of this intervention is appropriate. With regards to this aim, the authors are also vague as to what the intervention will be. They argue that the evidence based Western interventions are not feasible in this population, however, their alternative proposal of "ritual" is vague. Furthermore, how might "ritual" interact with the sleep homeostatic and circadian system to improve sleep. The investigators need to carefully consider what elements of "ritual" have the potential to positivity impact sleep and circadian rhythms.

Project 2: This project aims to show that three components of current sleep hygiene recommendations do NOT impact sleep. Having a no impact is not appropriate for an intervention study. Furthermore, there has been a lot of research around the components the investigators propose to measure (e.g., physical activity, screen time and food intake in the hour before bed) and most of these studies do not show a large impact of these measures. The authors also state that if these three components do improve sleep, it is unrealistic to implement such behavioural changes in the targeted population. Therefore, the benefit to the Māori will be limited. Furthermore, changing behaviour an hour before sleep is unlikely to have an impact.

Project 3: The most promising component of this project is the sleep ninja app that delivers digital CBT-I which has been shown to be highly effective in adults in western countries.However, I have the impression that this is already a funded project and studies testing the efficacy of Sleep Ninja are already underway. The evidence for the other intervention components is minimal.

Project 4: There is a lot of evidence that adolescents benefit from later school start times. While this intervention will likely be effective, it is not very novel.

Research impact **Score: 4**

The strength of this proposal is that interventions are tailored to the Māori, however, the interventions are either not novel or have been shown to be minimally effective in Western populations.

Some of the interventions, Sleep Ninja and delaying school start times are very likely to benefit youth in New Zealand.

Potential for outcomes **Score: 5**

By addressing sleep, this proposal is likely to impact other health related outcomes in youth and thus address inequalities. One of the limitations of the proposal is that the authors do not measure the impact of their interventions on other outcome variables, including physical health. This should be a secondary aim.

Expertise and track record **Score: 7**

The research team is exceptional. They have had great success running large studies and working with the Māori community. The team would benefit from great sleep expertise.

Collaboration and integration **Score: 6**

The team is well composed and includes stakeholders who will be able to inform policy change. This is perhaps most exciting with regards to school start times.

General comments

Applicant peer review report

Reviewer # 39

Proposal details

Title	Whakatipu Rakatira: improving sleep as a vehicle to grow healthy future leaders
First named investigator	Professor Rachael Taylor (University of Otago)

Rationale for research — Score: 7

This is a very strong application addressing a common issue across the life span - including infants and children - i.e. sleep and behavioral sleep problems that adversely impact health, mental health, quality of life and academic learning. Addressing sleep offers a non-stigmatizing approach to addressing inequity in health and healthcare and the suite of projects in this proposal makes use of existing workforces and programs in universal settings (well childcare, schools) that will enable equitable access to any potential benefits.

Design and methods — Score: 6

Each project has been carefully designed with 'gold standard' methodologies to answer their questions including a stepped wedge trial in schools and a MOST trial in teenagers. The interventions are being co-designed with end-users from the get go which is likely to enhance engagement and uptake. The trials use a combination of objective and subjective outcome measures which is key to sleep research where subjective measures alone have been criticized for bias. All projects are addressing contemporary gaps in knowledge. I do have some concerns about the feasibility of recruiting the sample size and conducting projects 1 and 3 on time and would like to see evidence to support feasibility. I do also wonder why they have not included a health economics evaluation for all trials as this could strengthen their case for roll out should any intervention prove effective.

Research impact — Score: 6

This suite of projects has huge potential for impact across children and their caregivers because: (1) sleep problems are very common; (2) sleep problems have documented adverse flow on effects across health, mental health, and school engagement and learning; and (3) the methodologies chosen are gold standard and thus the evidence coming from these trials will be publishable in high impact journals and will likely lead to international keynotes and changes in national and international guidelines. Further, the proposal has included early and mid-career researchers and 4 PhD stipends that will help grow capacity in future sleep research and trial methodology in New Zealand.

Potential for outcomes — Score: 6

The potential for positives outcomes is excellent. This is because the interventions have been designed to work in "real world" settings, making use of existing, universal workforces, and will be co-designed to fit with existing workforce flows - essential to any uptake of effective interventions. In addition to strong trial design methodology (so we can trust the outcomes), the team have excellent, pre-existing avenues for outcome translation in healthcare, policy and education. These avenues are stronger in health than in education, but I note formative work for the schools' trial is underway and includes representatives from the Ministry of Education.

Expertise and track record Score: 6

This is an internationally renowned team with strong and deep expertise in all aspects of paediatric sleep, Māori research and strong experience in the conduct of trials in community settings. They have excellent and relevant international collaborators on sleep and the MOST methodologies.

Collaboration and integration Score: 5

Generally strong collaborations esp with international partners and community collaborators. Many of the team have worked together before with strong outputs so appear well integrated. They have good governance structures for the projects.

I would like to see collaboration with a health economist to document the cost-effectiveness of the interventions. I would like to see the collaboration with the education researcher consider including learning/academic outcomes for the school trial, over and above school absences.

General comments

Applicant peer review report

Reviewer # 62

Proposal details

Title	Whakatipu Rakatira: improving sleep as a vehicle to grow healthy future leaders
First named investigator	Professor Rachael Taylor (University of Otago)

Rationale for research — Score: 6

A really detailed and coherent rationale has been provided for the project, and includes key areas which would be expected to determine the rationale for this series of studies. An area that would warrant some further discussion is the importance of a developmental perspective to justify why sleep interventions may differ across the life course, and why the expected outcomes may differ based on this.

How will learnings from previous sleep interventions designed in international contexts, be integrated into the design and implementation of the interventions described?

Design and methods — Score: 7

Project 2 - Justification for 'Three days will provide sufficient data to determine any impact on sleep'. It may be better to have a slightly longer period (maybe 2 weeks) for each stage to enable families to implement the condition and for wash out from the previous period to occur. There may be differences in when the 30-minute condition is conducted in the 1 hour, as well as the preceding 30 minutes might influence the final hour. Is it likely that doing 30 minutes exercise 1 hour before bedtime may have a different influence than doing closer to bedtime?

Project 3 - Although the sleep quality measure is well recognised, measures that capture sleep quantity, disruption and consistency would also be important. Other suggested measures could also be the EPOCH (which includes connectiveness but also other domains of wellbeing in adolescents and is well validated). Sleep can also have an impact on student cognition, so a measure such as the TEXI would also add to an understanding of addressing sleep.

Project 4 - In addition to duration and wellbeing, impacts on 'out of school' factors would be another interesting aspect to evaluate. For instance, their ability to engage in casual employment.

Research impact — Score: 7

The project has the potential to provide novel insights as to how sleep problems can be addressed in a number of different settings and age groups. Some further information as to how the learnings from each study can be used to contribute to the methodology and outcomes of the other projects would further highlight the research impact.

Another important consideration for all the intervention studies is the cost effectiveness/benefit of the interventions. WIll these be examined to enable an understanding as to the resource requirements for the interventions. For instance, changing school start times may have significant resourcing implications in terms of staff costs, resource availability as well as implications for social and community factors as well.

Potential for outcomes **Score: 6**

The series of research studies is clear and logical, with each study being feasible to conduct during the intended timeline. All projects include building on existing relationships and research partnerships. This would also help with the dissemination of the findings to inform practice and system change. Some more information about how findings may be implemented in the future beyond the studies would be helpful. For instance, if shown to be effective, will schools be able to continue to offer later school start times if shown to be effective in improving sleep outcomes, or will consideration also be given to other social and economic factors?

Expertise and track record **Score: 7**

The research team, and their contribution to the different projects, are exceptional. Their track record, current collaborations and expertise signifies they will be able to conduct the described projects to a high standard but will be able to use the findings to directly influence service and school practice related to supporting infant, child and adolescent sleep in a number of different contexts.

Collaboration and integration **Score: 7**

The project involves existing and new partnerships and collaborations with schools and early childhood services. The engagement with each as described in the studies shows a strong understanding of the importance of these collaborations to promote future adoption of the interventions, if shown to be effective.

General comments

The research team have described a series of really important studies which will further advance how sleep problems in New Zealand children are identified and addressed.

Applicant peer review report

Reviewer # 78

Proposal details

Title	Whakatipu Rakatira: improving sleep as a vehicle to grow healthy future leaders
First named investigator	Professor Rachael Taylor (University of Otago)

Rationale for research **Score: 6**

Solid rationale and impetus for this project including the linkage of sleep to diet, weight, school performance, injury, resilience, and mental health, and the indication that one-quarter of preschoolers and almost one-half of adolescents get less than recommended sleep in this region. The study findings will have implications both nationally and internationally for infants, children, and teens and their parents, as well as for healthcare providers and schools. Maori culture is included both in terms of the composition of the study team and in the co-design and partnership approach throughout each project, especially with consideration of incorporating cultural mores/traditions into project 1 to promote healthy infant sleep and for pre-bedtime routines. The 5 strands of Maori culture are woven throughout the projects, in particular recognizing that the common sleep hygiene recommendations from international groups conflict with Maori cultural practices. The MOST and the stepped wedge designs are innovative for this context; for example, the application states that of 84 registered MOST behavioral trials none look at sleep intervention in adolescents.

Design and methods **Score: 5**

The milestones appear carefully designed and feasible to accomplish within the 5-year period. Project 1 uses a co-design approach to design a sleep intervention with Maori providers, a key strength for feasibility and sustainability. Project 3 uses a MOST design and Project 4 uses a stepped wedged design, both of which are innovative and efficient to identify the active ingredients to optimize the intervention. The mixed methods approach that adds qualitative interviews of students, parents, and schools will provide rich context for interpreting the data in Project 4 and identifying potential untapped opportunities.

For all four projects, the sample sizes have adequate power grounded in scientific literature and a recognition of anticipated attrition. There should be a discussion of how to deal with missing data since that is likely to be an issue in these projects.

Project 1- enrolling 450 parents of newborns and 38 healthcare providers is a high volume for recruitment and retention. It would help to see evidence of the team's experience with similar scale trials and reassurance that the healthcare providers and the parents will be willing and interested to join the study. The primary outcome is parent-report of infant sleep, so discuss how this is subject to bias and if there's any opportunity to collect objective data such as with an accelerometer. A 20% dropout is included and the power calculations look appropriate. There are several implementation measures which will help with sustainability. Project 1 would benefit from preliminary evidence of efficacy/effectiveness of similar trials, especially given the shift away from international recommendations for sleep hygiene to better align with Maori culture.

Project 2- This is a provocative project in that it questions the utility of banning screens, food, and exercise prior to bedtime in children. It uses a strong randomized crossover trial that is balanced for order effects. How will the time of sleep be standardized within and between participants? Particularly for eating dinner or the other activities that may not typically occur in the child's daily routine prior to bedtime. It is a strength to allow families to choose 3 consecutive days every week to do this, but should there be standardization for weekend vs. weekdays or planned analysis of the potential differences between these two parts of the week? This is important considering wake time usually varies a lot between weekday vs. weekend day. The use of the body camera is a creative and objective way to monitor protocol compliance. Is the wrist-worn accelerometer a valid measure of sleep (and of MVPA and sedentary time) in kids? This is very important to demonstrate given this is the primary outcome. Are there other ways sleep will be measured beyond duration such as sleep latency, wake periods, etc. A sample size of n=60 seems feasible but the length of the study with the crossover design to require

some time commitment and discipline -- is there evidence of prior trials of the investigators that have achieved similar adherence and attrition? It seems there are many other variables that may impact results such as other kids in the household, household chaos, what foods/how much is consumed, what type and intensity of activity is performed, what total screen-time and MVPA the child typically gets.

Project 3- This is an innovative MOST design that will yield important findings on the specific features/components that do or do not matter for improving sleep disturbances in adolescents. n=320 is a large sample size especially since presumably the parent needs to be involved to provide consent (and they're part of the intervention to be "coaches" to their child), but this is an online, smartphone-delivered intervention so less participant burden and more aligned with adolescent preferences than coming in-person. Offering evidence of prior success with this type of participant recruitment and retention (<20% attrition) in an online trial would help reassure feasibility. This study (and the others) are clearly designed with the end-user in mind.

Project 4- Best example of preliminary prior published data to support hypothesis, as well as formative work including qualitative data from adolescents and parents and other key informants. Will a primary barrier be parents' work start times, or is the culture for adolescents to stay home and then catch a bus to school or drive themselves? The feasibility of the stepped wedge design is still something to consider given the impacts on the family/parents -- will some adolescents need to report to school at normal time or wake up at normal time because of household/family responsibilities or when transportation is available?

Research impact — Score: 6

The impact is clearly stated for each project, including improving healthcare providers' recommendations for infant sleep in Project 1, providing public health messaging for families of school-aged children in Project 2, interventions delivered digitally for adolescents with sleep disturbance, and school policies for school start times in older adolescents. There is also potential to impact policies and national professional organizations at a macro level.

Potential for outcomes — Score: 5

The milestones indicate the team's ability to publish findings throughout the 5-year project period as they are available for each individual study. More rationale is needed for project 2 why screen-time would impact sleep but not exercise or food consumed. Young Maori investigators are included with appropriate mentorship plans. There are extensive training opportunities for students and fellows. Collaborators/advisors are included to provide specific technical or analytic expertise. There is potential for economic outcomes specifically cost savings if these projects improve children's health and reduce medical expenditures, injuries, etc. It is not clear how project 4 school start time might negatively impact the economy as it relates to parents' work commitments.

Expertise and track record — Score: 6

This is an inter-disciplinary, experienced team that offers diverse expertise to ensure the successful completion of the 4 proposed studies. The PI Rachael Taylor has extensive prior funding from HRC and other funders so has lead multi-disciplinary teams and multiple research projects of similar large scale. Expertise includes biostatistics, sleep, mental health, physical activity, qualitative research, and Maori/cultural experts. Each project has preliminary or ongoing data collection that provides the foundation for this next trial/hypotheses. For example, there is an n=600 trial with 3 Maori health providers on the efficacy of a sleep intervention on infant sleep and caregiver well-being that has identified gaps such as more online resources and supports to increase adoption and sustainability. There is a recognition that providers should be part of the selection and adaptation to local needs to ensure sustainability.

Collaboration and integration **Score: 7**

Excellent inter-disciplinary, highly qualified team with a clear 4-person leadership structure for study governance and complemented by expertise in biostatistics, sleep measurement, sleep modification, school policy changes, and inclusion of Maori researchers and those with evidence of community-engaged research. Record of prior publications and relevant experience including prior/active formative trials. Sufficient FTE allocated to completing the proposed research.

General comments

This is a very well written and well conceptualized series of studies.

Applicant peer review report

Reviewer # 85

Proposal details

Title	Whakatipu Rakatira: improving sleep as a vehicle to grow healthy future leaders
First named investigator	Professor Rachael Taylor (University of Otago)

Rationale for research **Score: 6**

This is an ambitious project essentially divided into four different projects. The projects focus on different age ranges and /or different interventions within those age ranges.

The projects are well-structured, carefully planned and the references are comprehensive and accurate.

The authors are entirely correct that paediatric sleep medicine has evolved to focus on sleep disorders or illness, but there is a dearth of evidence to support how to improve 'sleep wellness'. Furthermore, as the authors explain, an intervention that might be acceptable to one group of adolescents with certain cultural expectations, might not be acceptable to another.

This research is undoubtedly important and worthwhile for New Zealand and will also carry weight internationally.

Design and methods **Score: 6**

The combined projects are ambitious, yet feasible and the outcomes which are well considered are achievable within the timeframe. Judicious use of methodology like multiple optimisation strategy, first described in Trials in 2019 is a thoughtful strategyto maximise the chance of finding appropriate and effective interventions.

The project designs are appropriate and importantly based on the considerable experience of the investigators who have track records at implementation within New Zealand.

Before reading this proposal I knew little about a culturally appropriate methodology and responsiveness to Māori. I have been educated during the reading of this proposal that goes so much further than the occasional 'lip service' seen in other international studies - this project truly co-creates, embeds and upholds Te Tiriti o Waitangiby reflecting Te Tiriti principles in every piece of work outlined.

Research impact **Score: 6**

Sleep underpins and cross-cuts virtually every aspect of well-being and health care. Early intervention is known to be effective and a focus on infant sleep, childhood sleep and adolescent sleep is welcome.

This project describes a credible pathway for how the projects may I influence policy, practice, and health servicesin NZ, leading to improved health or other social/economic impacts.

The research team all have credible experience and describe steps to maximise the likelihood of impact.

There is appropriate responsiveness to Māori.

Potential for outcomes Score: 6

The proposed research has potential to improve sleep and sleep related wellness/health knowledge and includes a clear focus on addressing inequalities.

Opportunities to strengthen health research workforce capacity for Māori and young investigators are well-described as are potential influences on policy, for example school start times.

.

Expertise and track record Score: 6

The team have a fascinating and entirely appropriate background or training, qualifications and experience,

I have looked up the publications and ongoing grant applications of applicants and these corroborate their role on this project. There is goodproductivity and delivery on their previous research funding, and there is appropriate responsiveness to Māori.

Collaboration and integration Score: 4

The team is excellent, but true collaborative integration across each project will be challenging . The proposed research reads as four discrete projects and at times it is difficult to understand how they will interface during the timespan of the project. For example, the team evaluating an intervention promoting Rongo time and Uru time for infants will have little foreseeable integration with the team evaluating the Sleep Ninja App for adolescents or the team assessing the impact of changing school start times for adolescents.

General comments
